# Survey of patients' view on functional split of consultant psychiatrists

**DOI:** 10.1186/1472-6963-13-362

**Published:** 2013-09-27

**Authors:** Millia Begum, Keith Brown, Anthony Pelosi, Jim Crabb, John McTaggart, Caroline Mitchell, Everett Julyan, Tony Donegan, Michael Gotz

**Affiliations:** 1 Hairmyres Hospital, NHS Lanarkshire, East Kilbride, Scotland G75 8RG; 2 Forth Valley Royal Hospital, NHS Forth Valley, Larbert, Scotland FK5 4WR; 3 Stobhill Hospital, NHS Greater Glasgow & Clyde, Glasgow, Scotland G21 3UW; 4 Crosshouse Hospital, NHS Ayrshire & Arran, Kilmarnock, Scotland KA2 0BE; 5 Wishaw General Hospital, NHS Lanarkshire, Wishaw, Scotland ML2 0DP

**Keywords:** Functional split, Inpatient psychiatric care, New ways of working

## Abstract

**Background:**

The functional split model of consultant psychiatrist care for inpatients has been one of the major service redesign that has occurred in the NHS in the last decade. It is unclear if this new split model offers any advantages over the previous sectorised model of working. More recent evidence has suggested that patients, carers and professionals have varied views regarding the benefits of this model.

This survey of patient’s views on models of consultant working is the first in Scotland and we have attempted to include a large sample size. The results suggest that after providing sufficient information on both models, the majority of patients from various Scottish health boards have opted for the traditional sectorised model of working.

**Method:**

During a four week period consecutive patients across 4 health boards attending the General Adult consultant outpatient clinics and those who were admitted to their inpatient ward were offered a structured questionnaire regarding their views on the functional split versus traditional sectorised model. Space was provided for additional comments. The study used descriptive statistical measures for analysis of its results. Ethical approval was confirmed as not being required for this survey of local services.

**Results:**

We had a response rate of 67%. A significant majority (76%) of service users across the four different health boards indicated a preference for the same consultant to manage their care irrespective of whether they were an inpatient or in the community (Chi-squared = 65, df = 1, p < 0.0001). In their unstructured comments patients often mentioned the value of the therapeutic relationship and trust in a single consultant psychiatrist.

**Conclusions:**

Our survey suggests that most patients prefer the traditional model where they see a single consultant throughout their journey of care. The views of patients should be sought as much as possible and should be taken into account when considering the best way to organize psychiatric services.

## Background

Burns has pointed out that most, if not all, of the recent radical changes in British psychiatric practice have been introduced with little public scrutiny or discussion [1]. Any debate that has taken place has largely been between professionals with scant consideration of patients’ wishes and experiences. This is true of the inpatient/outpatient functional split in the duties of consultant general adult psychiatrists. In the traditional approach (sectorised model), a single consultant provides both hospital and community care to a designated geographical catchment area. In this model, the consultant psychiatrist is the key link between the inpatient multidisciplinary team and the community mental health team. More recently, functional split model with dedicated inpatient and community consultant psychiatrists have been introduced. This model has followed on the establishment of various other functional teams such as early intervention in psychotic illnesses, assertive outreach, and crisis resolution and home treatment teams. In mental health trusts where this model has been adopted, consultant psychiatrists are no longer responsible for patients across the range of treatment settings [2]. A significant number of health boards have adopted the functional split model whilst the traditional model is still retained by few others.

There is disagreement amongst professionals about the merits of the newly introduced split model [1-5]. More recently, Singhal and colleagues sought the opinions of staff, service users and carers regarding this model. They conducted a small, semi-qualitative study at 3 NHS sites in England. Of the forty-three patients who responded to the survey, almost 65% were not aware of the changes in their service. Approximately half of service users did not like the change, similar proportion was satisfied to some extent and a minority was highly satisfied or extremely unsatisfied. About two thirds of the carers who responded were against the change. Although their quantitative data suggested that opinions were equally divided, the authors further commented that ‘most of the descriptive responses reflected dissatisfaction unlike the quantitative data’. One of the most consistent views they highlighted regarding the split model was that ‘continuity of care, the therapeutic alliance, the doctor-patient relationship and trust would be adversely affected, and this would in turn affect many other aspects’ [2].

## Method

Patients were recruited from the caseload of eight general adult consultant psychiatrists from four health boards across rural and suburban areas of Scotland. One of the consultants solely had inpatient duties within a split model. The remainder worked in a traditional sectorised model.

Consecutive service users attending the consultant psychiatrist outpatient clinics or those who were admitted under their care over a four week period were informed of the questionnaire. The front page explained the purpose of the survey and description of the two models as follows:

‘There are two different systems in operation currently in different parts of Scotland with how consultant psychiatrist work. In some areas, the same consultant psychiatrist is in charge of your care whether you are in hospital or if you are seen in the outpatient clinic. The consultant may have other doctors in his or her team (for example junior doctors) but he or she is the person responsible for your care no matter where you are’

‘In other areas, a different arrangement operates. In this model, your care will be managed by one consultant psychiatrist in the community but, if and when you get admitted to the psychiatric inpatient ward, your care will be taken over by another consultant psychiatrist until you are discharged back’

‘There are differences of opinion about which system of care is a better way of providing good patient care. Each system may have its own advantages and disadvantages. Surprisingly there are few attempts to ask patients what kind of arrangements they would prefer. That is what we hope you may be able to help us with’

‘It is up to you whether you answer this questionnaire or not. To maintain your privacy the questionnaire is anonymous. The number on the form allows us to track how many questionnaires have been handed out and cannot be linked with your name. Your care will not be affected in anyway if you choose not to take part.’

The questionnaires were handed to the patients by their consultants. Responses were handed back at the reception desk or to ward nursing staff, maintaining anonymity. Those who were considered to lack capacity to consent to taking part in the survey by their consultants were excluded based on their clinical judgement.

The standardized data collection form included a description of the background of the survey (as detailed above) and a series of 7 forced choice questions. Amongst the forced choice questions were demographic details, length of contact with the psychiatric services, type of services involved, number of hospital admissions experienced to date and time since their last inpatient admission.

Specific questions regarding preference of model were asked. These included options of (A) care provision by the same consultant who managed care in the community or (B) given by a different consultant responsible only for inpatient care or further two more options of (C) being unsure or (D) making no difference to them. Additional free space was given to provide reasons for this and further additional space was provided for any other comments (See Additional file 1).

Confirmation was received from Multicentre Research Ethics Committee that their approval was not required for this survey.

## Results

Out of 379 patients, 255 patients responded to the questionnaire during the four-week survey period (67% response rate). Of the 255 patients surveyed, 49 were inpatients and 206 were outpatients. The demographics data and service usage of the responders are shown in Table 1.

**Table 1:** Demographics data, illness characteristics and service use of the responders

Total number of respondents	255
Mean age (SD) of respondents (years)	43.8 (12.6)
Gender of respondents Male: Female	112: 143 (44% versus 56%)
Length of contact in months (SD)	10.3 (10.6)
Previous experience of:	
Inpatient ward	166 (65%)
Outpatient care	234 (92%)
Crisis/Home treatment team	103 (40%)

Of the 81 eligible service users who did not complete questionnaires the following reasons were provided; refused (n = 30), either doctor or patient forgot (n = 38), and no stated reason (n = 13). A further 43 were ineligible as they were too unwell or too distressed to engage in the survey.

Over three-quarters (195/255) of service users preferred their care to be given by the same consultant who managed their care in the community if they ever had to be admitted to hospital. 12% (31/255) of patients stated that it made no difference to them. Another 9% (24/255) were not sure and only 2% (5/255) preferred to have a different consultant responsible for managing inpatient care. There was a significant difference between the group that preferred the sector model (n = 195) versus those who did not (n = 65) (Chi-squared = 65, df = 1, p < 0.0001). User preference of service delivery is shown in Table 2.

**Table 2:** Service users’ preferences

	**Same consultant**	**Different consultant**	**Not sure**	**No difference**
Inpatients at time of survey (N = 49)	33	3	6	7
Outpatients at the time of survey (N = 206)	162	2	18	24

Using Fishers exact test with patients preference as a dichotomous outcome (same or different consultant), our results did not differ significantly between genders or those who had previously been exposed to inpatient care or not. We further analysed the data for the subgroup of 12 inpatients who had experienced an episode of inpatient care under one of the consultants working in the split model of care. Ten of these patients (83%) wanted the same consultant to look after their inpatient and community care in future. A further 68% of respondents provided additional reasons for their choice in the free space provided. Of the 195 patients who wanted the same consultant model, 75% (148/195) commented on reasons for their response. About half of those who felt it made no difference to them, (16/31) provided additional comments. Of those who supported the split model 4 out of the 5 and of those who were unsure 7 out of 24 also added comments.

Selected examples of patients’ comments which were supportive of the traditional model, supportive of the split model and those who had open minded views regarding either of the models are shown below in Tables 3, 4 and 5 respectively.

**Table 3:** Examples of patient’s comments in preference of the traditional model

	
•	‘I would prefer to see the same consultant in hospital, as I trust her already and would find it hard to build the same level of trust with someone else’
•	‘Continuity of care- New consultant may not read all information or interpret it differently’
•	‘A relationship of trust with your own consultant takes time to build and could never be done with a stranger that you meet now and again’
•	‘I’d prefer the above mainly for comfort and familiarity. I’ve shared my condition and stories enough times; having to repeat everything is pointless and counterproductive. It feels like an exercise in futility’
•	‘Continuity of care. I feel it would be best for me to have my care managed by someone who is familiar with me and can compare my symptoms with when I am well’
•	‘Over the past 5 years, I have had annual review as an outpatient and have seen a few different psychiatrists each time. I would prefer to be seen by one psychiatrist and also if admitted to hospital to see the same one’
•	‘Seeing the same doctor means you do not have to repeat your illness every time as this can be upsetting’
•	‘On one hand having the same consultant might give a sense of security. On the other hand, a different consultant having slightly different experiences, may also be a good thing-My personal view is that I would rather choose to have the same consultant’

**Table 4:** Examples of patient’s comments who were supportive of the split model

	
•	Because I have had 2 or 3 different consultants over the years
•	May prefer consultant in inpatient care rather than consultant that was involved in care in community

**Table 5:** Quotes from ‘open minded’ verbatim comments

	
•	‘It doesn’t matter who the consultant psychiatrist is as along as the standard of care does not diminish’
•	‘If I was mentally ill again, I would hope to be treated and cured as quickly as possible and I would not mind who treated me’
•	‘I don’t think it matters because the doctor at the time would still be reviewing my records and contact with other doctors, mental health nurses and Community Psychiatric Nurses’
•	‘In the United States, I was given a different consultant for inpatient care; this adversely affected the continuity of my medication/treatment due to different opinions. Medication was started inpatient and immediately stopped by my own consultant who understood my needs in more detail. I think if a different consultant is responsible only for inpatient care, it is important that they work in collaboration with the community consultant’

## Discussion

In this survey, a significant majority of the respondents indicated that they would prefer the same consultant to manage their care if they had to be admitted to hospital. A small minority of 2% specified that they preferred the split model. About one tenth of the respondents were unsure and a similar fraction felt it made no difference to them. The users were sampled in a systematic, pre-defined and consistent manner from a number of geographically varied urban and semi-urban services across Scotland. The results did not differ significantly between services. Both inpatient and outpatients were included.

One of the main limitations of the survey is the relatively small number of patients who have experienced functionalized care model. It could be argued that the majority of the respondents in this survey have only experienced sector care model and therefore lack the wider knowledge of the positive and negative impacts of alternative models of care. To ameliorate this, written information on the principal differences between the two models was provided to all respondents to assist them in making an informed choice. In addition to this, three-quarters of those with experience of the split model did not appear to support it although their number was small.

Another limitation of this survey was that the patients were from consultant caseloads only. Respondents might have established therapeutic alliance with their respective consultant with some degree of continuity in their care as opposed to the non-consultant caseload. However, patients in this survey were mostly middle-aged with an average of 10 years of involvement with mental health services and the majority had previous experience of inpatient care. Given their fairly longstanding involvement with services, they may be thought to be better placed to judge the proposed effects of service redesign. On the other hand, these patients may be more resistant to change from a familiar model.

For the minority of our service users who had open minded views regarding either models, their choice appeared to be based on the hope that good quality of care would be provided by either of the consultants. They also hoped that consultants would communicate well with each other in these circumstances.

The changes in the consultants model of working appear to have triggered by concerns raised by various policymakers (6,7,8,9,10). In 1998, The Sainsbury Centre for Mental Health published the findings of their survey highlighting poor standard of inpatient care. The issues were mostly related to lack of multi-disciplinary input into mental health wards and need to improve accommodation and staffing [6]. A further survey was carried out in 2004 offering a snapshot of inpatient services for the same year and the conclusions were based on questionnaires, sent to chief executives of NHS mental health trusts and acute ward managers in England. They received a 60% response rate. Amongst the various staffing and resource issues, the survey highlighted the need for effective leadership of acute inpatient wards from ward managers and consultant psychiatrist. They found that 144 wards (48%) did not have a ‘lead consultant’ [7]. In addition to this, the Royal College of Psychiatrists were concerned that consultant psychiatrists were carrying excessive caseload and were stretched between inpatient and outpatient services [8]. The New Ways of Working (NWW) initiative as part of revising overall staff roles and responsibilities in mental health teams saw ‘functional models’ as one way forward to address this. Specialist inpatient consultant psychiatrists were quoted as an example of NWW [8,9]. However, the authorities also acknowledged that disruption in continuity of care would be a challenge within the functional model [9]. To address this problem, one of the proposed solutions offered was to employ care coordinators who would ensure continuity [8].

Although, the underpinning principles of the NWW are unlikely to be challenged by most psychiatric professionals, the guidance does not give a clear evidence based argument for proposing functional split model of care as a possible solution. It was asserted that the mental health trusts where they have implemented the ‘functional model’ have been ‘very successful with demonstrable improvements in the inpatient experience for service users, carers and staff’ [9]. However, since the introduction of the functional split model, there has been little or no evidence to suggest that the change has significantly improved inpatient care or quality of consultants working over the traditional model of care. In a more recent study, Laugharne and Pant surveyed 26 NHS trusts in England whose inpatients participated in a previous Care Quality Commission survey of 2009. Their primary objective was to determine inpatient satisfaction with psychiatrists in mental health trusts with dedicated inpatient consultants versus those in trusts with sector consultants. Questions concerned were about respect, trust, being listened to and getting adequate time with their consultants. Although all factors except one, did not reach statistical significance at a 5% level, the trend was consistently in favour of the sector model of care [11].

The quality of patient experiences throughout their journey of care should no doubt be one of the most important factors in determining the best possible model of working for consultants. Improving inpatient care through good leadership is at the heart of this. The Royal College proposed the provision of dedicated ‘lead consultants’ for inpatient care who can provide expert input into key matters of inpatient service delivery, staff support, decision making and the overall acute care service coordination [10]. Based on this proposal, one possible model is to appoint ‘in-patient lead clinicians’ rather than dedicated in-patient consultants. This may address the problems faced within inpatient wards whilst preserving continuity of care. It will be important to distinguish between the concepts of clinical leadership and clinical responsibility within this model. These ‘lead clinicians’ could either be senior consultants with management experience or those who have special interest in acute in-patient care. Views of patients regarding this alternative model should be taken into account prior to any further changes.

## Conclusion

In the light of the above findings, we would agree with Burns that, in relation to the introduction of the functional split model, “the absence of debate in the professional journals is remarkable for what is potentially one of the most significant reorganizations of UK community mental healthcare in the last decade [1]. It would appear that large-scale changes in the organization of services have either taken place, or are being planned, against the wishes of those that use them. If their views are sought, patients may disagree strongly with planned developments.

## Competing interests

The authors declare that they have no competing interests.

## Authors’ contributions

MB, KB and AP conceived and designed the study, MB, KB, JC, JMcT, CM, EJ, TD, MJ collected the data. JC conducted the statistical analysis and all authors interpreted the results, drafted the manuscript and approved the final manuscript.

## Pre-publication history

The pre-publication history for this paper can be accessed here:

http://www.biomedcentral.com/1472-6963/13/362/prepub

## Supplementary Material

Additional file 1 Firstly, we would be grateful if you could give us the following background information. Click here for file
