# Supplementary material for: Survey of patients' view on functional split of consultant psychiatrists
Source: BMC Health Serv Res. 2013 Sep 27;13:362. doi: 10.1186/1472-6963-13-362 (PMC3849881; doi:10.1186/1472-6963-13-362)
Supplement: Additional file 1 — Firstly, we would be grateful if you could give us the following background information. [file 1472-6963-13-362-S1.doc]

**Additional file 1**

**Firstly, we would be grateful if you could give us the following background information:**

Your age: ______ Gender (please tick as √): Male

Female

For approximately, how long have you

had contact with psychiatric services for? ___________________

What contacts have you had with the psychiatric service?

(Please tick all that apply)

Inpatient ward

Outpatient Psychiatric Clinic

Crisis Team and/or Home Treatment Team

Have you ever been admitted to the psychiatric inpatient ward? If yes, approximately how many inpatient admissions have you had so far?

0

1-5

More than 5

When was the last time you were an inpatient?

Within the last year

1-5 years ago

More than 5 years ago

If you ever had to be admitted to hospital, given one choice, would you prefer your care: (Please tick)

(A) Be given by the same consultant who managed your

care in the community.

1. Be given by a different consultant responsible only

for inpatient care.

(C) Not sure.

(D) It would make no difference to me.

Please give reasons for the above answer

**Any other comments**
